# Investigating video consultations as a new form of care for neuropalliative patients in specialized outpatient care: results from the project TANNE (telemedical answers to neurological inquires in real time)

**DOI:** 10.3389/fneur.2026.1730210

**Published:** 2026-04-15

**Authors:** Christiane Weck, Stefan Lorenzl, Kirsten Brukamp, Viacheslav Galchenko, Tizian Juschkat, Christian Maier, Sebastian Müller, Jan Reichmann, Felix Tirschmann, Jürgen Zerth, Daniela Adolf

**Affiliations:** 1 Hospital Agatharied GmbH, Hausham, Germany; 2 Institute for Palliative Care, Paracelsus Medical Private University, Salzburg, Austria; 3 Research Group on Health, Technology and Ethics, Protestant University of Applied Science Ludwigsburg, Ludwigsburg, Germany; 4 MEYTEC GmbH Medizinsysteme, Werneuchen, Germany; 5 University of Bayreuth, Bayreuth, Germany; 6 ZEQ AG, Mannheim, Germany; 7 Catholic University of Eichstätt-Ingolstadt, Eichstätt, Germany; 8 StatConsult GmbH, Magdeburg, Germany

**Keywords:** hospice, neuropalliative care, specialized outpatient care, symptom control, telemedicine, video consultations

## Abstract

**Background:**

Currently, there is no standardized care for neuropalliative outpatients in Germany. Those who require specialized palliative care often receive it from a Specialized Outpatient Palliative Care (SOPC) team or hospice, but without regularly access to neurological expertise. The TANNE project elaborated hints for a cost-effective telemedical link between neuropalliative expertise (a Neuropalliative Telemedicine Center) and existing comprehensive palliative care structures such as SOPC team and hospice. Video consultations enable a joint assessment of symptoms and targeted therapy. The TANNE project aims to ensure that patients with neurological diseases or neurological symptoms in the palliative phase receive professional neurological/neuropalliative care at home or in hospices in a resource-efficient manner.

**Methods:**

A prospective, partly-cluster-randomized, two-arm intervention study with a delayed-start design was conducted between May 2021 and June 2023. The intervention group received video consultations whenever neurological problems occurred (event). The control group continued to receive treatment as usual. Primary endpoint was the change in symptom burden (iPOS – Integrated Palliative Outcome Scale) measured intra-individually before and after an event. Various secondary endpoints were assessed, namely patient’s general well-being and patient’s and professional’s satisfaction with treatment.

**Results:**

A total of 32 teams participated, recruiting 114 patients and registering 77 events. The primary endpoint showed a reduction in symptom burden of 2.6 (±4.15) points after teleconsultation, compared to 1.3 (±8.36) points in the control group (not statistically significant on a 5 percent level). This reduction was more pronounced in the ‘Psychological and Practical Problems’ subscale. High satisfaction scores with treatment and care were found in the intervention groups among patients and professionals.

**Discussion/Conclusion:**

The teleconsultation evaluated in the TANNE project represents a form of interaction between neuropalliative expertise and specialized palliative care (SOPC team, hospice) that has not existed in this form and scope before. Due to insufficient number of cases in combination with teams’ rejection to participate and additional withdrawals the projected primary endpoint could not be satisfied on a statistically relevant level. Nevertheless, results provide reliable points of reference for further research on the support of decision-making processes within SOPC and hospice teams in neurological cases through targeted teleconsultation services.

## 1 Introduction

People with advanced neurological diseases, particularly neurodegenerative conditions, can benefit from palliative care (1, 2, 3, 4, 23, 24). Palliative care for people with neurological diseases such as for example amyotrophic lateral sclerosis (ALS) requires extensive specialist knowledge of neurology (5, 2). These diseases can cause symptoms similar to those experienced by other palliative patients, such as dyspnea and pain. However, they also frequently experience specific neurological symptoms, such as painful spasticity, swallowing disorders, pseudohypersalivation, epileptic seizures and myoclonus. The symptom burden is considerable and complex, making palliative care necessary (4). For this reason, palliative care for these patients is now routinely required, in some cases from the time of diagnosis (e.g., for patients with amyotrophic lateral sclerosis) (2). Currently, there is no systematic approach to the palliative care of neurological outpatients (6, 23) in Germany. Patients requiring specialized palliative care are subsequently cared for by a Specialized Outpatient Palliative Care (SOPC) team or in a hospice, with no access to neurological or neuropalliative expertise within the framework of standard care. In some cases, patients cannot be admitted to existing care structures (SOPC teams and hospices) due to a lack of expertise in dealing with these clinical cases. Due to the rarity of many neurodegenerative diseases, neurologists also lack the expertise to provide palliative care for these conditions. A pilot project has successfully demonstrated the feasibility of providing telemedical support for neurological patients in specialized outpatient palliative care (7).

To provide an *ad hoc* care model with specialist neurological expertise embedded in organizational structures, the TANNE project was launched with funding from the Innovation Fund (01NVF19004). As part of the project, specialist neurological consultation services for SOPC teams and hospices were tested and evaluated. In addition to consultation and specialist support, the project included training and further education opportunities. This form of care focused on neuropalliative teleconsultations, which could be integrated into the care processes of SOPC teams and hospices, regardless of their location. To provide professional and organizational support, a neuropalliative telemedicine center was established at a Bavarian hospital. All hospices and SOPC teams in Bavaria were offered the opportunity to participate in the project via email or telephone. The center provided a 24/7 on-call service so that SOPC teams and hospice carers could contact with neurological queries. Audiovisual teleconsultations were carried out via the mobile tele-system (MEYDOC® Client). Regular webinars and online training courses were held for medical staff and carers on current topics and frequently asked questions (see Supplementary material S1). Public relations work in the form of lectures served to promote the acceptance of integrating palliative medicine into the treatment of neurological diseases.

The TANNE project was based on the following working hypothesis: *Adding neuropalliative expertise to the care of people with neurological diseases or symptoms in outpatient palliative care improves the recognition and targeted treatment of distressing symptoms, measured by iPOS – Integrated Palliative Outcome Scale - at an intra-individual level in intervention and control arm to elaborate average treatment effect of the treated and average treatment effect of controls* (8). Hence, an average treatment effect was planned to finally highlight the inter-group developments in time.

The paper at hand refers to the dimension of medical effectiveness. It aims to demonstrate the extent to which the symptom burden of people with neurological diseases in the palliative phase can be alleviated through the addition of neuropalliative expertise.

## 2 Materials and methods

### 2.1 Study design and time points of data collection

The project’s study design was published in Gatter et al. (9) comprising the adaption on the idea of a complex intervention (10). TANNE comprises the structure of a complex intervention within complex systems (11), especially recognizing various relationships within and between SOPC teams. The project was comprehensively evaluated considering multidimensional aspects of outcomes in the line of a complex intervention that had to be reflected involving quantitative and qualitative surveys (10). The evaluation assessed the effects of medical efficacy and satisfaction with the form of care, as well as health economic aspects, particularly the impact on induced services.

A two-arm, prospective, partly cluster-randomized approach with a delayed start design was chosen for TANNE.

Employing a cluster randomization address both effects published in literature for choosing that kind of organizing a sample (12). TANNE wanted to address a group sample – the SOPC/hospice representing the complex intervention environment – as well as the necessity for being strict considering ethically-based question differentiating teleconsultation use within the same SOPC/hospice group. Moreover, it was not feasible for an SOPC/hospice team to conduct a teleconsultation for only some of the patients in its care and treat the others independently of the findings of the teleconsultation, as this would inevitably lead to carryover effects.

The S1 study arm was connected to the telemedicine center from the onset. The teams were equipped with the necessary technology (camera package). The second arm (S2) was initially observed (standard care; S2.1 = control group (CG)) and was connected to the telemedicine center after 12 months (intervention; S2.2 = delayed intervention group).

Randomization to either treatment or control arm was applied to the hospice/SOPC teams in a 1:n ratio and all patients of a team were treated according to the team’s assignment because of the change within second arm from standard care to delayed intervention group. As the intergroup comparison between the intervention and control groups corresponds to the comparison of the S1 and S2.1 study arms, i.e., all patients in the intervention group S1 with a neurological problem within 24 months are compared with those in the control group S2.1 with a neurological problem within the first 12 months, randomisation is performed at a ratio of 1:2. Two additional aspects have to be mentioned. As SOPC and hospice teams can vary greatly in composition and size, randomization was additionally stratified by hospice/SOPC. Additionally, during a pilot project (7) some of the participating SOPC teams were already provided with camera kits as part of a feasibility study, which gave them the opportunity to ask neuropalliative questions via video consultations or other methods. Seven teams had already participated in the preliminary study and used video consultations. These teams were assigned to the intervention group in the TANNE study, assuming a probability of 1 to be allocated to treatment arm. In consequence, to guarantee balancing randomization was applied to the remaining SOPC teams (first stratum) in a 1:3 ratio. Participating hospices – the second stratum - were randomized to the intervention and control group in a 1:2 ratio.

These adoptions – employing a partial randomization approach – ensure that group sizes were balanced despite the fact that SOPC teams were already included in the teleconsultation arm and that hospices have comparatively fewer patients. Block randomization with a random block length of 4 or 6 was performed by a statistician, who was not involved in the intervention.

The evaluation perspective mainly focused on summative evaluation however some aspects of a formative, process-orientated evaluation was additionally targeted, namely aspects of perceived satisfaction with treatment mode asked for the stakeholders involved (13).

A crucial item in TANNE evaluation refers to the term “event”. That term reflected an altered anticipation of information about care receivers` consideration about the occurrence of a neurological problem by involved caregivers. One event corresponded to a teleconsultation in the intervention group, while in the control group an analogous event was used in which the patient, had he been in the intervention group, would have received a teleconsultation. Hospital admission always corresponded to an event, both in the intervention and control group. To identify suitable patients, both teams (IG, CG) were given a list of diseases, symptoms, and medications that could trigger a teleconsultation (see Supplementary material S2). This list of symptoms was generated based on prior experiences from the pilot trial (7). The final decision that an event exists was made by the teams in both groups. In the control group, an “event” was considered to have occurred if a specific neurological problem arose that could have been addressed through a teleconsultation, or if additional assistance was sought to treat the patient, e.g., literature research or consultation with practicing neurologists. Each reported consultation equivalent was checked by the study office.

Considering summative evaluation, time points for data collection during the study were:

1) At inclusion: demographic data.
2) Before the event (consultation/consultation equivalent): disease stage analogous to palliative phases.
3) Before the event (from −3 to 0 days) and after the event (from 3 to 7 days). The following were collected at these time points: iPOS, overall well-being, Eastern Cooperative Oncology Group (ECOG) as a clinical performance measurement and diagnoses.

### 2.2 Primary endpoint

The primary endpoint was based on the validity and high relevance of the individual parameters described in the literature. The integrated Palliative Care Outcome Scale (iPOS) was therefore selected as the primary endpoint (14). The iPOS is a short, multidimensional instrument for assessing the symptoms and problems of palliative care patients over the previous 7 days. It measures changes in individuals before and after a teleconsultation, or in the control group before and after the onset of neurological symptoms.

The total iPOS score can range from 0 to 68. In addition, three subscales are calculated: “Physical Symptoms, Gastrointestinal Symptoms, Psychological And Practical Problems” [range 0–32], “Gastrointestinal Symptoms’ [range 0–12], and “Psychological and Practical Problems” [range 0–24] (15). In TANNE the iPOS was assessed from the perspectives of professional caregivers (physicians and nurses), relatives and patients, with the professionals' iPOS being used for interpreting the primary endpoint. In consequence, an intra-individual change before and after the event was measured and assessed by professionals (doctors and nurses) from the relevant teams. To sketch an average treatment effect, group comparisons of intra-individual changes were performed using a mixed linear model, in which patients’ affiliation to an SOPC or hospice team was modelled as a random effect due to cluster-randomization (with underlying compound symmetry correlation structure).

Sample size estimation was based on an assumed group difference in intra-individual changes in iPOS of 4.3 with a standard deviation of 9.3 (14). Applying to a significance level of 5%, 75 patients with an event per group (S1.1 and S1.2 over 24 months, and S2.1 over 12 months) are required to reach 80% power.

### 2.3 Secondary endpoints/further data collected

One of the secondary endpoints was information on patient’s overall well-being (7-point Likert scale). An other secondary endpoint was the assessment of individual items on satisfaction with treatment and care (also measured on a 7-point Likert scale). Further data collected included the ECOG performance status, diagnoses, patient demographic data and an assessment of disease stage (analogous to the assessment of palliative phases in specialized palliative care) (16).

## 3 Results

### 3.1 Recruitment and documented events

Specialized outpatient palliative care structures (SOPC teams and hospices) were recruited between October 2020 and the start of the study. The clinical study was conducted from 15.05.2021 to 30.06.2023. The Intervention group S1 was connected to the telemedical center from the start of the study; the control group S2.1 had treatment as usual in the first year (S2.1) and was connected to the telemedicine center in the second year (S2.2). Thus, on the 1st of June 2022, the control group switched to the delayed intervention group (S2.2).

**Figure 1:**
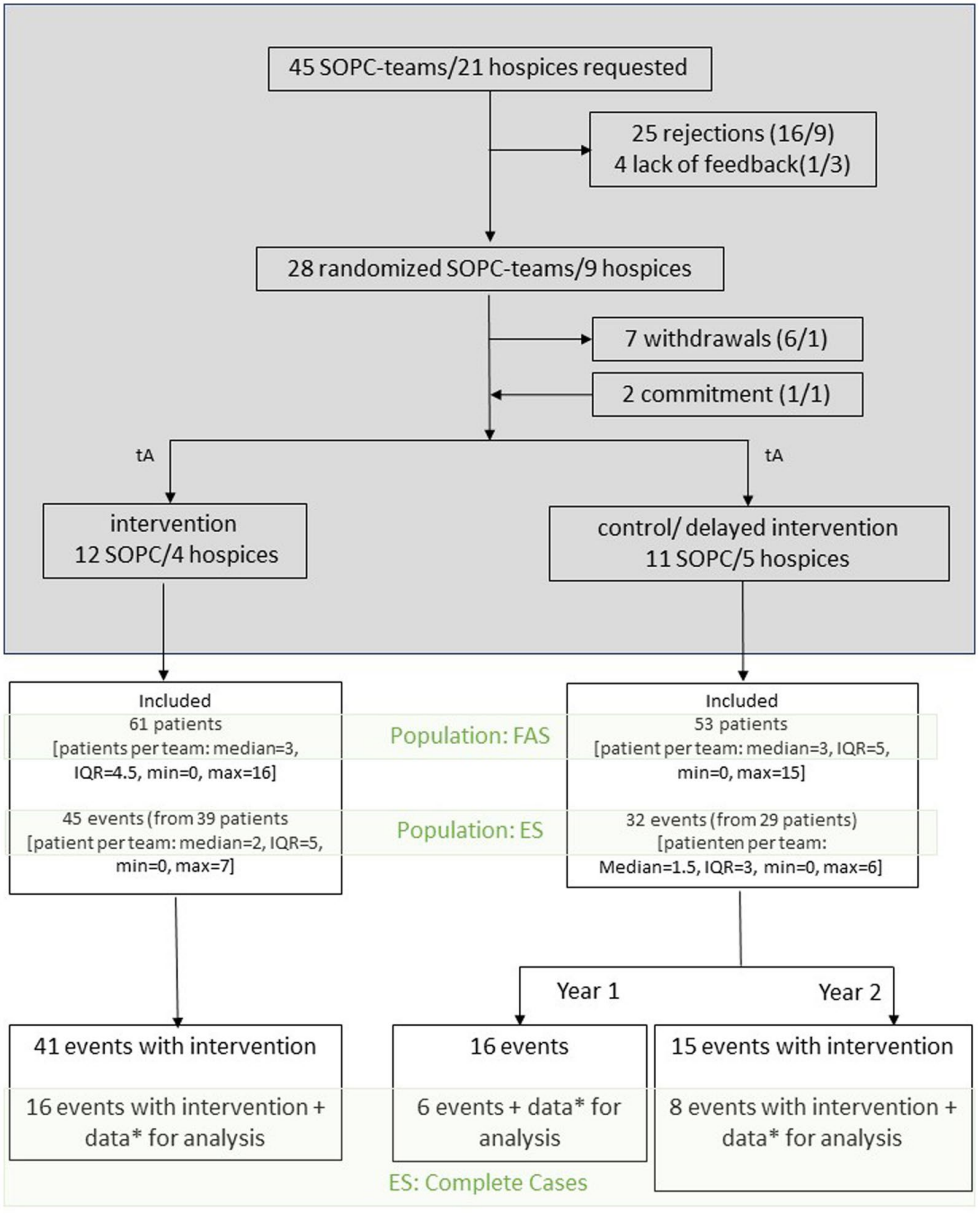
Flowchart: Upper section (shaded in grey) showing the recruitment process of SOPC teams/hospices (start of study tA), with the number of SOPC teams/hospices (SOPC/hospices) in brackets. Lower section: Depiction of the analysis populations (highlighted in green: FAS: Full Analysis Set, ES: Evaluation Set), included patients and events (either hospital admission or teleconsultation or treatment as usual in the control group), those with teleconsultations (intervention) are shown in the bottom. *Data on the primary endpoint, iPOS complete at tv (before consultation) and t2 (after consultation) (professionals).

After agreeing to participate in the project, individual SOPC and hospice teams were assigned to the treatment or control group. During the project period, there were delayed acceptances and rejections from individual teams (see Figure 1).

**Figure 2:**
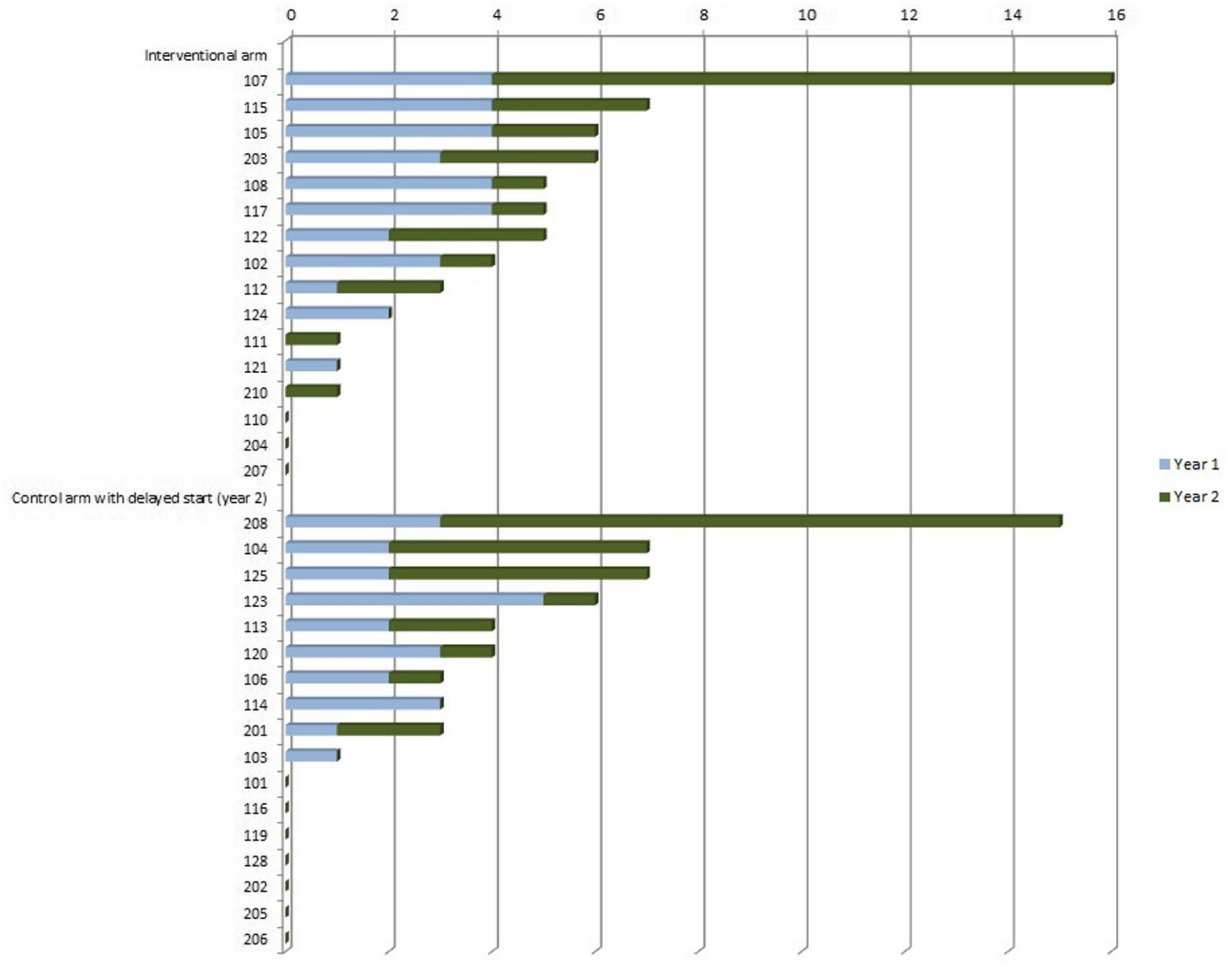
Number of patients recruited (*x*-axis) per team (*y*-axis, 1*xx* SOPC teams, 2*xx* hospice teams), separated by interventional arm and control arm (including delayed intervention group S2.2 – year 2), separated by study year.

Data from 16 teams in the intervention arm (S1.1 and S1.2) and 16 teams in the control arm (S2.1 and S2.2) were included in the evaluation. A total of 114 patients were recruited according to the inclusion criteria over the entire study period; a breakdown by study arm can be found in Table 1. Recruitment among the teams was highly variable, with one team including 16 patients - the highest number of patients. Ten teams did not include any patients (see Figure 2). The same patient could be included more than once if they developed a new neurological problem. This applied to a total of six patients. Five of these patients experienced a second event during the study period. One patient had a total of five events.

**Table 1:** Number of patients included per study arm, number of events per study arm (event corresponds to teleconsultation in S1 and S2,2; consultation equivalent in S2,1), one patient could also be included in the evaluation with several events.

Comparison group		Patients per study arm		Events per study arm
		*N*	%	*N*
Intervention group	S 1,1	32	28.1	25
	S 1,2	30	26.3	20
	Total (S 1)	61	53.5	45
Delayed intervention group	S 2,2	29	25.4	16
	Total (S 1 + S 2,2)	90	78.9	61
Control group	S 2,1	24	21.1	16

A total of 77 events (teleconsultations/consultation equivalents in the control arm or hospital admissions) were documented (see Figure 1 and Table 1). Of these, there were 41 events with teleconsultation in the intervention arm, 16 events (consultation equivalents/hospital admissions) in the control arm, and 15 events with teleconsultation in the delayed intervention arm.

The target number of cases was not reached in either group. It was decided not to extend the control arm before switching to the delayed intervention group, which constituted a protocol deviation. It is worth mentioning that some teams immediately withdrew from the project reporting that they did not perceive an direct benefit (i.e., no teleconsultation) and the associated documentation effort was reported as too high by those teams. Ethical concerns were also reported, as teams in the waiting period were unable to provide consultations, so extending the control arm was not feasible. Due to funding restraints it was not possible to recruit additional control teams. The study agreed on continuing collecting data with as many teams as possible, in order to gain hints not even for the primary endpoint but also for secondary endpoints and process evaluation.

### 3.2 Teleconsultations

Teleconsultations were generally pre-scheduled. On the outpatient side (SOPC team or hospice), the teleconsultation was accompanied by either a doctor and/or a nurse. In hospices, nurses were predominantly present, whereas in SOPC teams, doctors were predominantly present. The duration of the consultation varied depending on the issue at hand. On average, a teleconsultation lasted 26.3 (±11) minutes, excluding preparation and follow-up. Technical preparation and follow-up (i.e., setting up and dismantling the equipment) took an average of 9.6 (±7.1) and 7.4 (±4.9) minutes for the teams on site. At the telemedicine centre, this took 4.4 (±3.8) and 1.3 (±0.5) minutes, respectively. Report generation took an average of 10.25 (±2.8) minutes.

Table 2 shows which measures were implemented following the consultation. In most cases, oral medication was started or the dosage was changed. In fewer cases, non-oral medication was started or changed. Non-medicinal measures also accounted for only a small proportion of the consultation recommendations. Several recommendations were often made during one teleconsultation. In 53.4% of cases, the recommendations were implemented in full, in 25.8% of cases only partially, and in 5.2% of cases not at all.

**Table 2:** Measures following the consultation.

Comparison group		new oral medication started	existing oral medication changed	new non oral medication started	non oral medication changed	changings in non-pharmacological treatment
		N (%)	N (%)	N (%)	N (%)	N (%)
IG	S 1,1	14 (63.6)	14 (63.6)	5 (22.7)	3 (13.1)	1 (4.5)
	S 1,2	9 (56.3)	12 (75.0)	3 (18.8)	1 (6.3)	2 (12.5)
	total (S 1)	23 (60.5)	26 (68.4)	8 (21.1)	4 (10.5)	3 (7.5)
Del. IG	S 2,2	10 (66.7)	9 (60.0)	1 (7.7)	0	1 (6.7)
	Total (S 1 + S 2,2)	33 (62.3)	35 (66.0)	9 (17.0)	4 (7.5)	4 (7.5)
KG	S 2,1	3 (42.9)	4 (57.1)	0	1 (14.3)	2 (28.6)

### 3.3 Patient characterization

The demographic data of patients who experienced an event can be found in Table 3. Comparing the intervention group (S1) and the control group (S2.1) reveals several differences. Patients in the intervention group were numerically older than those in the control group. The average age of the intervention group was 72.1 years (± 14.9), compared to 61.2 years (± 10.0) for the control group. The ECOG status differed between the groups. At the time of the event, 84.2% of patients in the intervention group had an ECOG score of greater than 2, compared to 71.4% in the control group. Regarding disease stage, a numerically higher proportion of patients in the intervention group were deteriorating or dying compared to the control group (71.4% vs. 62%). Differences between the groups were also observed in the iPOS. A higher iPOS score indicates a higher probability of patients being in an unstable or deteriorating phase. Before consultation, the intervention group had a numerically higher iPOS than the control group (mean 28.4 (± 7.1) points vs. 25.6 (± 4.9) points). This difference in baseline values was also present among patients for whom intra-individual change could be observed, albeit to a lesser extent.

**Table 3:** Demographic data of patients with an event; sex/team type/region/underlying disease per patient, ECOG per event (multiple events per patient possible).

Socio-economic attributes	Patients'characteristics	Comparison group and study arm					
		Intervention group (IG)			delayed IG	IG total	Control group
		S1,1	S1,2	S1	S2,2	S1 + S2,2	S2,1
Sex (*n*; %)	Number of patients	23	17	39	15	54	13
	Missing	0	0	0	0	0	1
	Male	9 (39.1)	9 (52.9)	17 (43.6)	6 (40.0)	23 (42.6)	6 (46.2)
	Female	14 (60.9)	8 (47.1)	22 (56.4)	9 (60.0)	31 (47.5)	7 (53.8)
Age average (STD)	Missing	0	0	0	0	0	1
		73.1 (14.8)	69.6 (15.2)	72.1 (14.9)	68 (7.7)	70.9 (13.3)	61.2 (10.0)
ECOG	Missing	3	6	9	6	12	3
	ECOG≤2	3 (15.0)	3 (16.7)	6 (15.8)	6 (40.0)	12 (22.6)	2 (28.6)
	ECOG>2	17 (85.0)	15 (83.3)	32 (84.2)	9 (60.0)	41 (77.4)	5 (71.4)
Patient per team type	SOPC	21 (91.3)	13 (76.5)	33 (84.6)	12 (80.0)	45 (83.3)	14 (100)
	Hospice	2 (8.7)	4 (23.5)	6 (15.4)	3 (20.0)	9 (16.7)	0
Patient per region	Rural	8 (34.8)	7 (41.2)	15 (38.5)	14 (93.3)	29 (53.7)	11 (78.6)
	Urban	15 (65.2)	10 (58.8)	24 (61.5)	1 (6.7)	25 (46.3)	3 (21.4)
Underlying disease	Neurological	21 (93.1)	14 (82.4)	34 (87.2)	15 (100)	49 (90.7)	14 (100)
Neurodegenerative		15 (65.2)	7 (41.2)	21 (53.8)	10 (66.7)	31 (57.4)	3 (21.4)

#### 3.3.1 Neurological diseases

**Figure 3:**
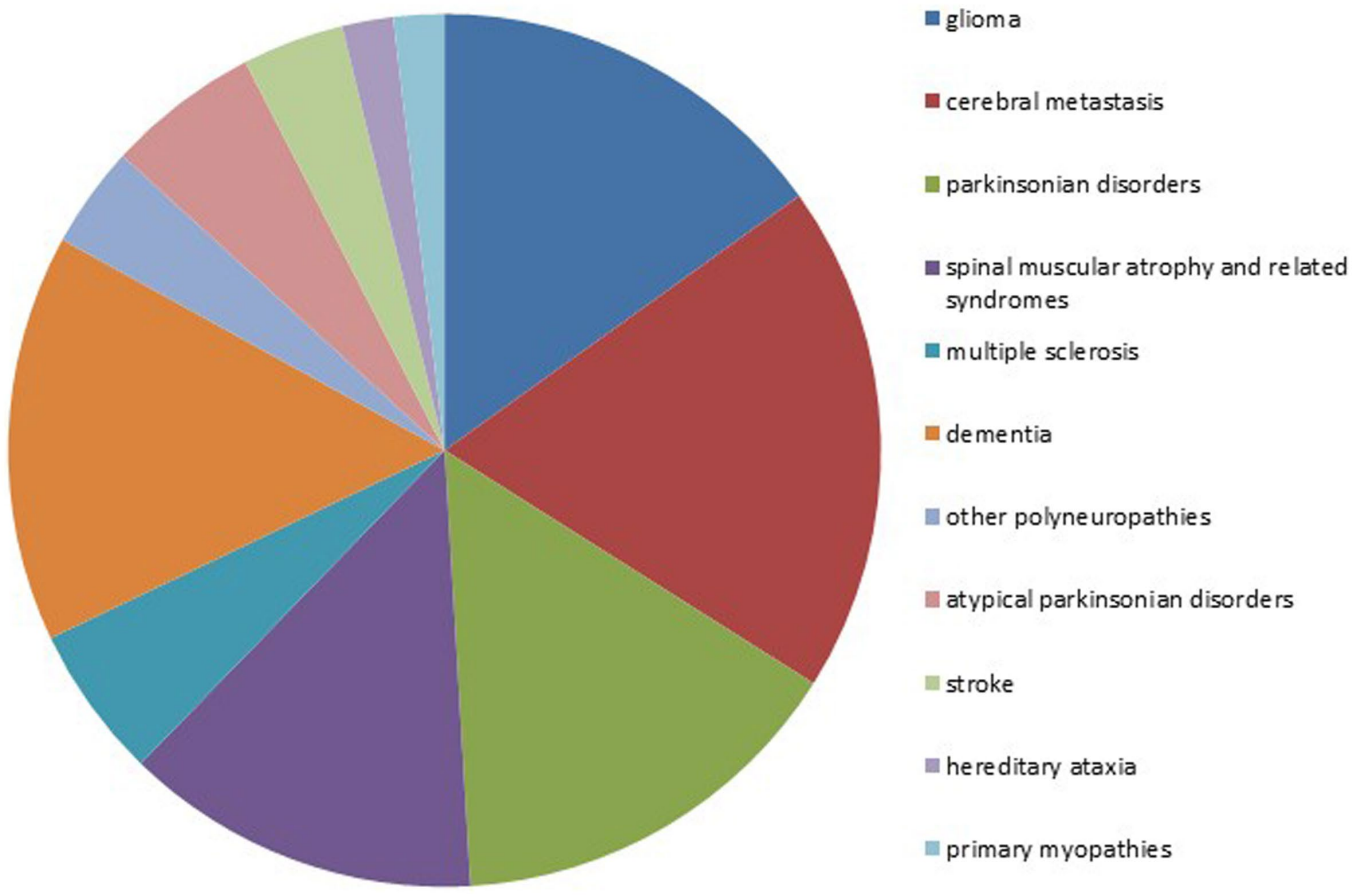
Overview of the neurological diagnoses of patients having an event.

Figure 3 shows the neurological diseases of the patients with an underlying neurological disease and an event recorded (in the intervention and control group). Of these diagnoses, 56.6% are classified as neurodegenerative diseases and 33.9% as neuro-oncological diseases. Patients who did not have a neurological disease but did have a neurological/neuropsychiatric symptom were also included in the study but are not shown in this figure.

#### 3.3.2 Symptoms of patients in the context of teleconsultation

**Figure 4:**
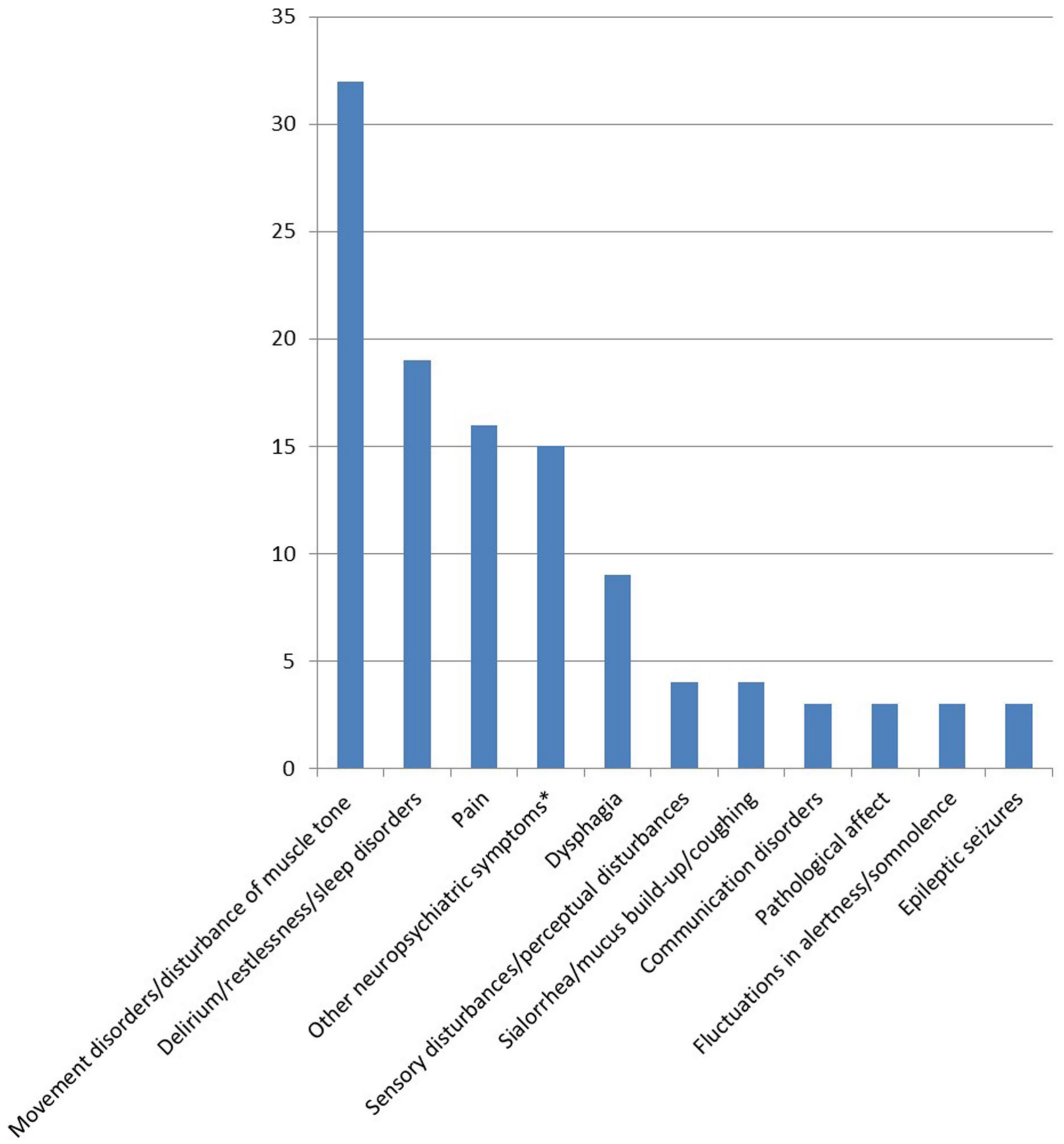
Overview of symptoms discussed in teleconsultations, harmonised form, documented by the consultant. *(Hallucinations, delusions, anxiety, aggression, panic attacks, mood instability).

As part of the teleconsultation, the consultant documented the symptoms of the patients in an open question. A total of 132 symptoms were recorded. Figure 4 provides an overview of these symptoms in a harmonized form. The most common group of symptoms relates to movement disorders (*n* = 30) and includes diagnoses such as increased muscle tone (*n* = 9), rigidity (*n* = 6) and akinesia (*n* = 4), as well as other symptoms including tremor, freezing, startle inhibition and dystonia (*n* = 1 each). Another large group of symptoms includes restlessness, delirious states, and sleep disorders (*n* = 19). The pain group includes unspecified pain (*n* = 10), pain associated with increased muscle tone/spasticity (*n* = 2), and pain associated with restless legs syndrome (*n* = 3). Fatigue/exhaustion, dry mouth and orthostatic dysregulation were each mentioned twice, while all other symptoms/issues were mentioned once each (double vision, choking spells, a runny nose, sleep apnea, weakness, dizziness, visual disturbances, suicide attempts, a risk of falling, a change in therapy goals, dry eyes, dyspnea, nausea/vomiting, supply problems and myoclonus).

### 3.4 Primary endpoint – symptom burden

#### 3.4.1 Main results

The primary endpoint was measured using iPOS, which is based on the change in symptom burden experienced by each individual before and after the consultation. Only events with complete data were included in this evaluation, employing a complete case approach (see Figure 1 for details on complete cases) (17). Sixteen data sets were used to calculate the primary endpoint in intervention group S1 and six in control group S2.1 (see Table 4). These were ultimately included in further analyses. These are questionnaires completed by professionals. As expected, we received the largest number of completed questionnaires from this group. Taking the complete case approach into account, seven complete data sets regarding patients’ view from the intervention group and two from the control group could be included. Additionally, nine complete datasets of relatives in the intervention group were available and one of that in the control group.

**Table 4:** Descriptive analysis of intra-individual change - iPOS (professional) – descriptive statistics, complete cases, tV, before consultation (−3 to 0 days before consultation); t2, after consultation (3 to 7 days).

iPOS (professionals)	“Pre event” (tV)		“After event” (t2)	
	IG	CG	IG	CG
	*N* = 16	*N* = 6	*N* = 16	*N* = 6
*N* (missing)	0	0	0	0
Average	27.8	26.2	25.1	24.8
STD	7.86	5.04	7.17	6.11
Min	15	20	14	18
Q1	2.,0	21.0	20.5	20.0
Median	26.0	26.5	23.5	24.0
Q3	33.5	30.0	28.5	28.0
Max	44	33	43	35
change to tV	—	—	*N* = 16	*N* = 6
N(missing)			0	0
average			−2.6	−1.3
STD			4.15	8.36
Min			−14	−13
Q1			−5.0	−9.0
Median			−1.5	0.5
Q3			−0.5	4.0
Max			3	9

Due to the low number of cases, we refrain from demonstrating any effect on a significant statistical level. The measured changes are reported purely descriptively.

Considering the change of iPOS in the intervention group we could highlight a decline of 2.6 (± 4.1) points whereas the decline in the control group can be summed up to 1.3 (± 8.4) points. To control for cluster randomization and baseline difference when estimating the average treatment effect these values were confirmed in the model estimation (see Supplementary material S3). However, the reduction of 2.6 points in the IG was not statistically significant: [−5.70; 0.44]. All sensitivity analyses yielded no statistically significant results. The results are represented in tabular form in Supplementary material S3. With respect to other stakeholders’ response the iPOS assessment done by relatives yielded similar assessments to those made by the professionals: changes of −1.23 [−9.92; 7.465] in the IG and 2.00 [−17.52; 21.52] in the CG. Unadjusted, the improvement in the IG was even more pronounced at −2.5 (± 5.0) points. With total iPOS scores of 31.4 (± 9.2) and 27.0 (± /), respectively, the data from relatives showed the highest value of the three evaluators (professionals, patients and caregivers).

#### 3.4.2 iPOS subscale – psychological and practical problems

The assessment of professionals referring to the ‘psychological and practical problems’ subscale, which ranges from 0 to 24 points, gave some interesting hints because of change in subscale of −1.73 points [−2.92; −0.54] on a statistically significant level (*p* = 0.007) see Table 5. However, the average treatment effect of the controls showed a decline of 0.569 points [−2.59; 1.45] but on non-statistically significant level. No significant difference was documented in the intergroup comparison either: −1.16 [−3.50; 1.18], *p* = 0.31 (see Supplementary material S3).

**Table 5:** Descriptive analysis of intra-individual change – iPOS subscale: psychological and practical problems (professionals) – descriptive statistics, complete cases; tV, before consultation (−3 to 0 days before consultation); t2, after consultation (3 to 7 days).

iPOS subscale: psychological and practical problems (professionals)	tV		t2	
	IG	CG	IG	CG
	*N* = 21	*N* = 7	*N* = 21	*N* = 7
*N* (missing)	0	0	0	0
average	9.9	11.4	8.1	10.9
STD	2.6	3.6	2.7	3.0
Min	3	5	3	7
Q1	8.0	8.0	7.0	7.0
Median	9.0	13.0	8.0	11.0
Q3	12.0	14.0	10.0	14.0
Max	14	14	13	14
change to tV	—	—	*N* = 21	*N* = 7
*N* (missing)			0	0
average			−1.7	−0.6
STD			2.6	1.9
Min			−7	−3
Q1			−4.0	−3.0
Median			−1.0	0.0
Q3			0.0	1.0
Max			2	2

#### 3.4.3 Missing data

Some missing values have to be reported, the existence of that missing data was caused by various circumstances.

Firstly, it is not valid to replace individual values in the iPOS, meaning that the total score cannot be calculated for partially completed questionnaires. Secondly, due to clinical deterioration, death, and so on, it was not possible to collect complete data in all cases. The table in Supplementary material S4 lists the missing iPOS total scores by time (before the teleconsultation, tV, and after the teleconsultation/equivalent consultation, t2). Also shown here is the mean iPOS score as a measure of symptom severity.

Initially, several methods had been planned for primary endpoint analyses, e.g., a mixed model over all timepoints including a baseline iPOS and further confounders, etc., but the amount of missing data was much higher than expected (for 64% of the events). That’s why, focus was on complete case analysis in which data of 16 and six events in the treatment and control group, respectively, could be used. Nevertheless, a multiple imputation (at least using predicted mean matching (18)) (*n* = 100) based on the groups, sex, age, level of care, and ECOG status was conducted for all events (45 in IG, 16 in CG) as a post-hoc analysis resulting in the estimates provided in Supplementary Tables S7, S8 with slightly higher intra-individual changes but not hints for an altered interpretation of the intervention results.

#### 3.4.4 Summary

In consequence, some descriptive declines in iPOS could not be finally interpreted as a valid effectiveness of the treatment scheme. Here, problems with statistical power as well as differences in the starting values of intervention and control group have to be considered. However, the descriptive results could be seen as a strong emphasis for a more expanded study scheme in further research strategies.

### 3.5 Secondary endpoints

#### 3.5.1 Overall well-being

Patients were asked to rate their overall well-being after the consultation using a Likert scale ranging from 1 (“much worse”) to 7 (“much better”) (see Table 6). Albeit the low level of answers to be interpreted (S1,1 N = 9, S2,1 N = 5) the description gives some hints for building a hypothesis getting in touch with a structured video-based consultation patients answers with a more propensity of being well. The sensitivity analysis also underlines the hypotheses having a clearer differences in the self-assed rating (difference of 1.35 [−0.58; 3.28] points) among intervention group patients who had fully implemented the consultation recommendations (see Supplementary material S5).

**Table 6:** Descriptive statistics Likert scale; 1 (“much worse”) to 7 (“much better”) – Overall well-being of the patient after consultation.

Comparison group and study arm		How have you been feeling overall since you contacted the doctors about this problem?								
		N	*N* (missing)	Average	STD	Min	Q1	Median	Q3	Max
IG	S 1,1	9	1	4.8	1.7	1	4.0	5.0	6.0	7
	S 1,2	3	0	3.7	1.5	2	2.0	4.0	5.0	5
	total (S 1)	12	1	4.5	1.7	1	4.0	5.0	5.5	7
del. IG	S 2,2	7	0	3.9	2.3	1	1.0	4.0	6.0	7
	total (S 1 + S 2,2)	19	1	4.3	1.9	1	3.0	5.0	6.0	7
CG	S 2,1	5	1	3.4	1.3	2	2.0	4.0	4.0	5

*N*, Number of patients.

#### 3.5.2 Some formative evaluation – patient’s and healthcare professional’s satisfaction

**Figure 5:**
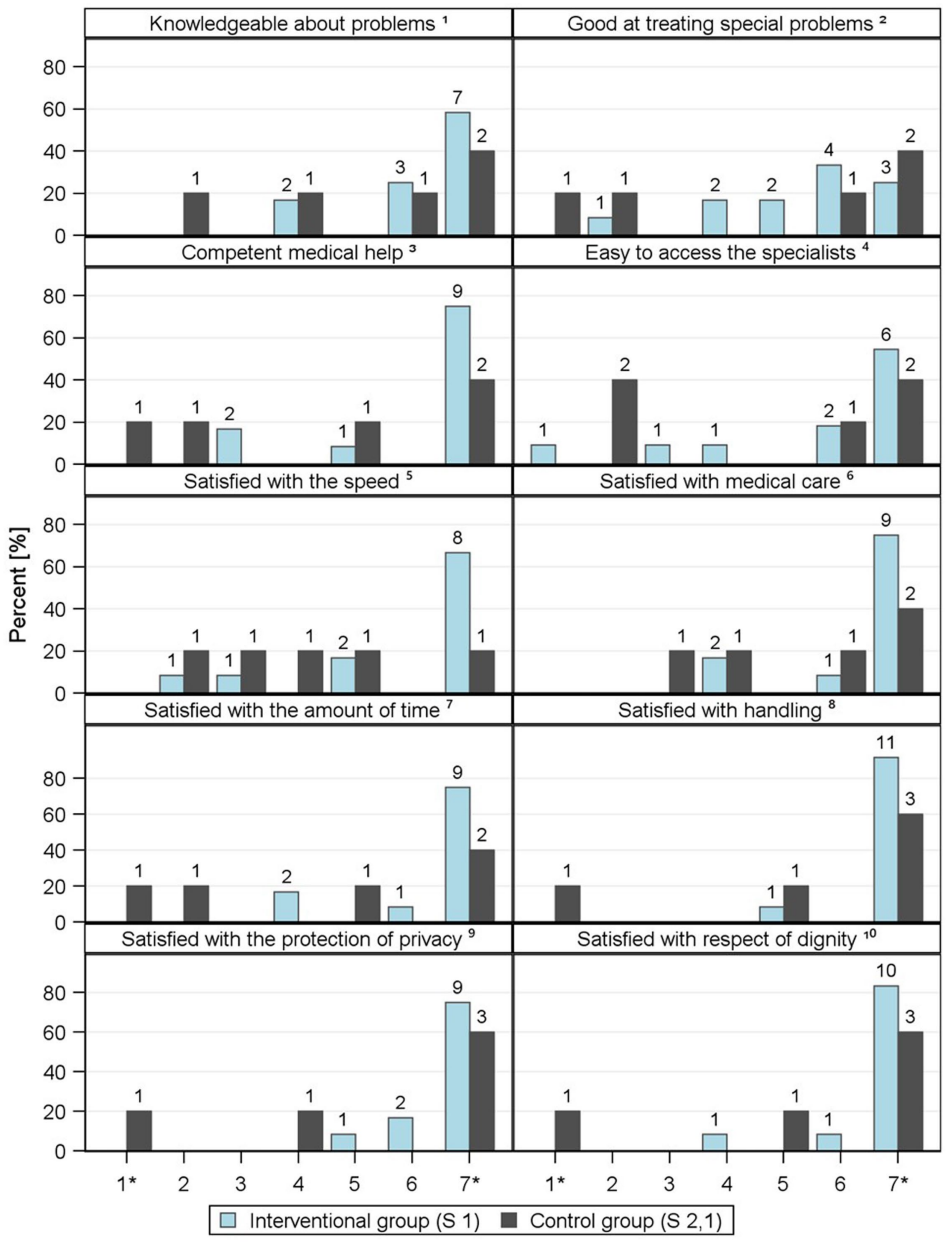
Illustration of individual items relating to ‘satisfaction with treatment/care’ in the form of frequencies, provided by patients. Scale from 1* - does not apply at all to 7* - applies completely ^1^The doctors seemed to be very knowledgeable about my medical problems. ^2^The doctors were also very good at treating special medical problems. ^3^I felt that I always received competent medical help whenever I needed it. ^4^I found it very easy to access the specialists I needed. ^5^I was very satisfied with the speed with which I received the appropriate medical help. ^6^I was very satisfied with my medical care overall. ^7^I was very satisfied with the amount of time the doctors took. ^8^I was very satisfied with the courtesy, respect, sensitivity, and friendliness of the doctors. ^9^I was very satisfied with the protection of my privacy. ^10^I was very satisfied with the way the doctors respected my dignity.

TANNE also evaluated the implementation of the *ad hoc* care model and its assessment by the involved stakeholders, focusing on its perceived practical utility. Here, various individual items were used to survey patient satisfaction with treatment and care and the results are shown in Figure 5. A comparison of patient satisfaction surveys reveals consistently high to very high satisfaction in the intervention group (Likert Scale 1–7, with 7 being the highest satisfaction). The mean values range from 4.2 to 7 points across all item groups in the intervention arm. Overall, the intervention groups consistently achieved higher scores than the control group, but the control group only consists of 5 individuals, this means that the comparison between the two groups can only be used to a limited extent.

The evaluation of time doctors spent with patients in the intervention group was reported by patients on satisfaction score of 6.42 (S1). Additionally, a score of 6.17-point was reported in the intervention group regarding the impression of receiving competent medical care. The mean overall satisfaction rating for medical care is 6.4 in the intervention group.

**Figure 6:**
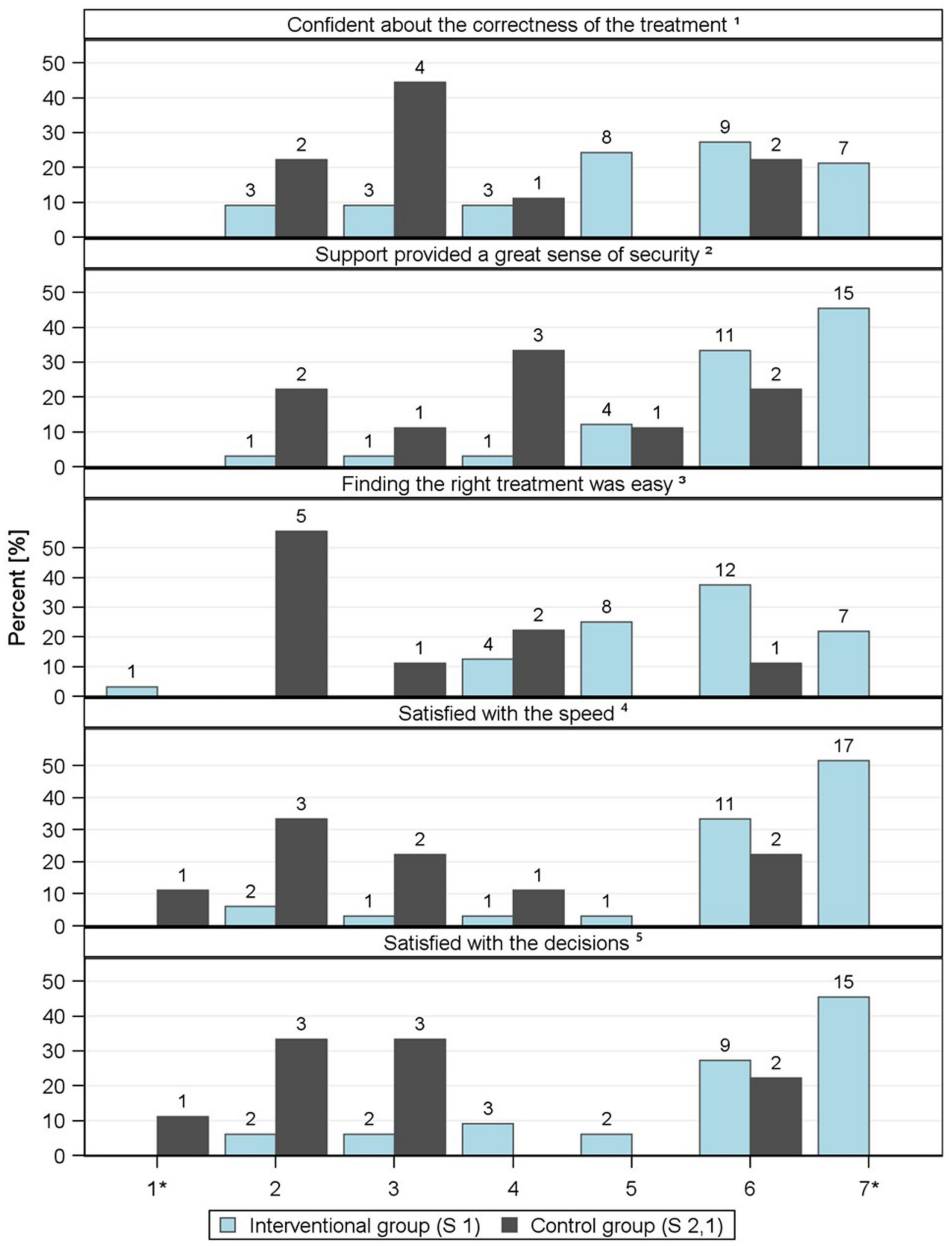
Illustration of individual items relating to ‘satisfaction with care’ in the form of frequencies, provided by professionals. 1* - does not apply at all to 7* - applies completely. ^1^I was very confident about the correctness of the treatment for the neurological problems. ^2^The support I received in solving the neurological problem and/or the additional resources I used gave me a great sense of security. ^3^I found it very easy to find the right treatment for the neurological problems with support or other sources. ^4^I was very satisfied with the speed with which the patient received the appropriate neurological therapy. ^5^I was very satisfied overall with the decisions regarding the treatment of his or her neurological problems.

A survey of healthcare professionals regarding satisfaction with care revealed similar impressions on TANNE. Healthcare professionals focusing their experience within the intervention groups showed high to very high confidence in the correctness (mean value 5.15) of the treatment decisions and a sense of security with (mean value 5.79) the treatment decision. In comparison, professional health carers rate their satisfaction about their own decision environment much lower, between values of 3.56 and 3.11, respectively. Items and distribution are shown in Figure 6. Only nine data sets can be used for the control group for this analysis, which makes it difficult to compare the two groups. We could not evaluate whether the self-assessed impressions were probably driven by any personal background factors of the professionals or the sole impact they got from the TANNE experience.

## 4 Discussion

The teleconsultation service implemented and evaluated in the TANNE project is an alternative way for neuropalliative medical experts to interact with outpatient or inpatient palliative care teams (SOPC and hospices). An intergroup difference respecting the intra-individual change in iPOS could not be demonstrated on relevant statistical significance. Here, the small number of complete cases have to be seen as the main limitation for the interpretation. With respect to the descriptive data we see continuous hints for an effective improvement in symptom burden in the intervention group in comparison to patients within the control group. Here, some similarities to published literature on validation of iPOS could be mentioned. Murtagh et al. (14) elaborated an improvement in symptoms in the intervention group, whereas there was no change in the control group. However, in contrast to Murtagh et al. (14) patients included in the TANNE frame are referred to a later stage of illness with greater severity among them. Furthermore, the limitation of a quasi-experimental design should be considered. Considering the patients selected within the study, the proportion of patients with neurodegenerative diseases was higher in the intervention group than in the control group at baseline. Neurodegenerative diseases are an example where possible changes resulting from medication adjustments require a longer observation period.

Sensitivity analyses revealed an influence of the different iPOS baseline values. For analyzing the impact of other covariates - as initially planned - a deeper investigation in further research is needed. In our study, those covariates reflecting the patients’ health status, e.g., diagnosis and ECOG status, were only examined within a subgroup analysis. Based on ECOG data and iPOS baseline values, it appeared that the intervention group was clinically more severely affected than the control group. According to the validation data of the iPOS, it was postulated that the symptom burden in the intervention group could improve by 4.3 points in an intra-individual comparison. As already mentioned, the patients in the iPOS validation data were in ‘earlier’ stages of the disease than those examined in TANNE (14). The effect size originally assumed in the study could not be demonstrated to this extent and must therefore be questioned. Methodologically, there was also no pure balance of patients in terms of severity. A particular challenge remains in selecting suitable standardized measurement instruments and measurement periods for a heterogeneous patient population with internal oncological and neurological/neurodegenerative diseases at various stages of the disease and care process.

A further relevant result for additional research on the effectiveness of TANNE-treatment stemmed from the results referring to “Psychological and Practical Problems” subscale. Our evaluation showed decline on the iPOS scale in the intervention group on statistically significant level. However, there was not a reliable result in the intergroup comparison due to the very small number of cases in the control group. Overall, the challenges in missing an appropriate number of cases reflecting the complexities of specialized palliative care influenced the planned test situation for the primary endpoint in more intensive matter as could be expected at the starting point of the study.

Especially, some heterogeneities in conducting documentation within the teams involved are worth mentioning. During the course of the study, it was observed that changes in the disease course (progressive/acute deterioration or death) resulted in questionnaires not being completed at certain time points. Taking the complete case approach into account also led to a lower number of evaluable cases, which were necessary to determine the complete intra-individual course of the disease. Additionally, there was marked patient and event heterogeneity, reflected in some covariates (e.g., age, ECOG), but this could only be modelled to a limited extent due to the number of cases. The small number of patients is a major limitation when evaluating all the results. We believe that one of the main reasons for this is the high workload of the teams (SOPC and hospices), which has been exacerbated by the ongoing pandemic. In summary, it should be noted that recent TANNE results give hints that the symptom burden questionnaire is only suitable for measuring intra-individual changes to a limited extent. For patients with neurodegenerative diseases (about 56.6% of the patients), the time period may be too short for changes to have become sufficiently effective.

Telemedicine has basically developed to a common method of providing comprehensive, widespread and specialized palliative care but mainly implemented on a project level rather than standard care. Many studies have demonstrated the feasibility and acceptance among stakeholders of a wide variety of telemedicine applications (19, 20). Several studies have also demonstrated its effectiveness (21). The main focus of TANNE was to find a suitable form of care for people with neurological diseases in palliative care. Telemedicine allows expert knowledge (neuropalliative expertise) to be combined with the existing comprehensive expertise of the SOPC teams in this area. This form of care supports existing outpatient palliative care structures.

Indications from the process evaluation and health economic analysis (not shown here) underline the hypotheses for a deeper research on neuropalliative teleconsultation strategies to be implemented in regular care environments because of potential effects of rationalizing induced resources as well as impacts on the level of satisfaction among all stakeholder within a SOPC or hospice environment (part of data not published yet). Considering the results of formative evaluation, professionals in the intervention group in particular stated answers feeling more confident in treating neurological patients thanks to the support provided by the neuropalliative teleconsultation service because of more assistance of applying the right treatment for the patients. Additionally, the quantitative health economic analysis indicated the potential to effectively reduce induced costs, primarily through a decline in emergency hospital admissions, and to extend the overall uninterrupted period in SOPC (data not yet published).

To sum up, there are some hints for reliable testable hypotheses derived from primary and secondary endpoints. Additionally, there are indications of positive perceptions among stakeholders involved in the care process, providing impetus for further investigation. Feedback from participating SOPC and hospice facilities, particularly from patients and their relatives, confirms the basic hypotheses regarding the usefulness and necessity of supportive telemedical neuropalliative counselling. It is important to continue addressing open questions regarding the fundamental effectiveness and cost-effectiveness of this approach, while also exploring the most suitable institutional setting for this new form of care.

## 5 Limitations

There are several limitations within the study. This is only a partially randomized study with seven teams that were assigned directly into the intervention arm based on previous experience. This approach was chosen to avoid distorting the control results by taking into account prior experience of dealing with neurological problems. During the study performed, implementation of teleconsultation could be seen in a sense of a learning system, whereby teams are able to solve recurring problems independently over time. Since the term ‘event’ is very broad, and the teams decide which patients to present and which not to, there is naturally a certain recruitment bias. However, this exists equally in both the intervention and control groups. Both arms were provided with decision aids to assist in patient selection. The iPOS collected by professionals was chosen as the primary endpoint, as we expected to receive the most complete questionnaires here. Of course, expectation bias can be a problem here. Ideally, the patients’ own data would be analyzed. If the patients’ data were analyzed, there would be seven data sets available in the intervention group and two data sets in the control group, while data from relatives would be available for nine (IG) and one (CG)patients. This means that, in this setting, the professional version must be used. A major limitation is that the patient population was heterogeneous, comprising both oncological and neurological/neurodegenerative diseases at very different stages of care. This, with small case numbers and thus no possibility of adjustment, makes it difficult to interpret the results concerning effectiveness. Particularly in the case of neurodegenerative diseases, multiple medication adjustments may be necessary, and a longer observation period would therefore be advisable. In the original study protocol, this was formulated with a supplementary survey date after 21 days (if necessary). Due to the very limited data collected at this point in time, this was not reported here. That specified time window was chosen for a very heterogeneous patient group consisting of largely stable patients to dying, rapidly deteriorating patients in order to detect an effect but also to prevent possible deterioration from progressing too far. The model of teleconsultation for SOPC/hospices was tailored to external settings resulting from the embeddedness into the Bavarian environment. To some extent, learnings from the team-related implementation towards teleconsultation may be transferrable to other regions within Germany. However, for additional research some investigation on aspects of palliative teams’ willingness to implement structured forms of teleconsultation should be conducted.

## 6 Conclusion

An intergroup comparison could not demonstrate clinical efficacy in terms of a reduction in symptom burden. However, several results suggest that this form of care should be evaluated more extensively. The basic idea behind this resource-efficient approach to care is to integrate neurological expertise into the non-clinical care of critically ill patients, minimizing stress for patients and their relatives.

Finally, it is important to identify potential open questions and institutional and organizational hurdles to the implementation of this form of care. Some of potential general barriers to telemedicine solutions mentioned in the literature may also apply here, namely (1) minimum equipment with a basic technological solution, (2) willingness to integrate it into the work process—for professional specialists—or into everyday life, (3) economic aspects of refinancing, and (4) currently inadequate organizational or institutional structures for implementation (22).

## Data Availability

The datasets presented in this study can be found in online repositories. The names of the repository/repositories and accession number(s) can be found at: https://innovationsfonds.g-ba.de/projekte/tanne.353.
