# Supplementary material for: Investigating video consultations as a new form of care for neuropalliative patients in specialized outpatient care: results from the project TANNE (telemedical answers to neurological inquires in real time)
Source: Front Neurol. 2026 Apr 15;17:1730210. doi: 10.3389/fneur.2026.1730210 (PMC13126451; doi:10.3389/fneur.2026.1730210)
Supplement: Supplementary file 1 [file Data_Sheet_1.DOCX]

# Supplement 1

# Webinar topics:

- Delirium
- Acupressure and acupuncture in neuropalliative care
- Cannabis
- Epileptic seizures in neuropalliative care
- Malignant brain tumours
- Case studies (amyotrophic lateral sclerosis)
- PEG tubes in neurodegenerative diseases
- Myoclonus
- Stroke in palliative care
