# Supplementary material for: Investigating video consultations as a new form of care for neuropalliative patients in specialized outpatient care: results from the project TANNE (telemedical answers to neurological inquires in real time)
Source: Front Neurol. 2026 Apr 15;17:1730210. doi: 10.3389/fneur.2026.1730210 (PMC13126451; doi:10.3389/fneur.2026.1730210)
Supplement: Supplementary file 2 [file Data_Sheet_2.DOCX]

# Supplement 2

# List of symptoms, diagnoses and medications as possible triggers for a teleconsultation/consultation equivalent:

| **Diagnosis** |
| --- |
| Motor neuron disease; (amyotrophic lateral sclerosis/progressive muscular atrophy/primary lateral sclerosis) |
| Glioblastoma (primary brain tumor) |
| Parkinsons disease |
| Atypical parkinsonian disorder (Multiple system atrophy, progressive supranuclear palsy, coritco basal degeneration) |
| Multiple sclerosis |
| Status epilepticus |
| Epileptic seizure/epilepsy |
| Cerebral metastases |
| Meningeosis carcinomatosa |
| Ischemia/stroke |
| Cerebral hemorrhage |
| Subarachnoid hemorrhage |
| Dementia |
| Creutzfeld Jakob disease/ prion disease |
| Chorea huntington |
| Meningitis |
| Polyneuropathia |
| Heriditary Neuropathies |
| Spinal Muscular Atrophy |
| Spino-cerebellar ataxia |
| Muscular dystrophies |

| **Symptoms** |
| --- |
| Delirium |
| Siallorhoea |
| Sleep disorders |
| Disorientation |
| Swallowing disorders |
| Laryngospasm |
| Intracranial pressure |
| Headache |
| Paresis |
| Dysarthria |
| Aphasia |
| Confusion |
| Tremor/myoclonus/spasticity |
| Pathological Laughter/crying |
| Mucus accumulation |
| Pain in neurological primary diagnosis |

| **Medication goup** | **Substances** |
| --- | --- |
| Antiepileptic drugs | Levetiracetam, lacosamide, valproic acid, lamotrigine, carbamazepine, oxcarbazepine, pregabalin, |
| Dompamine | Levodopa |
| MAO-B inhibitors | Rasagiline, selegiline |
| Dopamine agonists | Ropinirole, pramipexole, apomorphine, piribedil, bromocriptine, pergolide, lisuride, cabergoline, |
| COMT inhibitors | Entacapone, tolcapone |
| NMDA antagonists | Amantadine |
| Anticholinergics | Biperiden |
| ALS medication | Riluzole |
