# Supplementary material for: Investigating video consultations as a new form of care for neuropalliative patients in specialized outpatient care: results from the project TANNE (telemedical answers to neurological inquires in real time)
Source: Front Neurol. 2026 Apr 15;17:1730210. doi: 10.3389/fneur.2026.1730210 (PMC13126451; doi:10.3389/fneur.2026.1730210)
Supplement: Supplementary file 3 [file Data_Sheet_3.DOCX]

# Supplement 3

# Further analyses of the primary endpoint

**iPOS total score (professionals)**

| **Comparison group and study arm** | | **IPOS-Score** | | | | | | | | |
| --- | --- | --- | --- | --- | --- | --- | --- | --- | --- | --- |
|  |  | **N** | **N(missing)** | **Average** | **STD** | **Min** | **Q1** | **Median** | **Q3** | **Max** |
| IG | S 1,1 | 12 | 11 | 29.0 | 6.1 | 23 | 25.0 | 26.5 | 32.5 | 44 |
|  | S 1,2 | 13 | 5 | 27.8 | 8.1 | 15 | 23.0 | 26.0 | 37.0 | 39 |
|  | total (S 1) | 25 | 16 | 28.4 | 7.1 | 15 | 24.0 | 26.0 | 33.0 | 44 |
| del. IG | S 2,2 | 9 | 6 | 27.0 | 7.7 | 14 | 24.0 | 26.0 | 30.0 | 40 |
|  | total (S 1 + S 2,2) | 34 | 22 | 28.0 | 7.1 | 14 | 24.0 | 26.0 | 33.0 | 44 |
| CG | S 2,1 | 7 | 2 | 25.6 | 4.9 | 20 | 21.0 | 26.0 | 30.0 | 33 |

**Table 1: Descriptive statistics iPOS (professionals) before consultation/at event; IG… Intervention group, del. IG… delayed intervention group, CG…Control group**

|  | | | **N** | **Estimate** | **Lower** | **Upper** | **Pr > \|t\|** |
| --- | --- | --- | --- | --- | --- | --- | --- |
| Change - iPOS (professionals) | Comparison-group | IG | 16 | -2.625 | -5.694 | 0.444 | 0.086 |
|  |  | CG | 6 | -1.333 | -6.345 | 3.678 | 0.566 |

**Table 2: Statistical analysis of intra-individual change – iPOS (professionals); IG… Intervention group, CG…Control group**

|  | **Estimate** | **Lower** | **Upper** | **Pr > \|t\|** |
| --- | --- | --- | --- | --- |
| IG vs CG of Change - iPOS (professionals) | -1.292 | -7.168 | 4.585 | 0.635 |

**Table 3: Statistical analysis of the intervention for iPOS (professionals)**

| **Comparison-group** | **Estimate** | **Lower** | **Upper** | **Pr > \|t\|** |
| --- | --- | --- | --- | --- |
| **Change - iPOS (professionals)**  IG + del. IG vs. CG | -0.458 | -6.158 | 5.242 | 0.866 |
| IG (restricted to first year) vs. CG | 0.048 | -9.088 | 9.183 | 0.989 |
| IG + del. IG (with consultation recommendations fully implemented) vs. CG | -0.608 | -6.083 | 4.867 | 0.810 |
| IG vs. CG; adjusted to age and baseline | -0.622 | -6.022 | 4.779 | 0.797 |
| IG + del. IG vs. CG; adjusted to age and baseline | 0.178 | -4.699 | 5.055 | 0.938 |
| IG (restricted to first year) vs. CG; adjusted to age and baseline | 1.658 | -12.717 | 16.033 | 0.669 |
| IG + del. IG (with consultation recommendations fully implemented) vs. C9G; adjusted to age and baseline | 0.066 | -5.289 | 5.421 | 0.978 |

**Table 4: Statistical analysis of the intervention for iPOS (professionals) – sensitivity analyses; IG… Intervention group, del. IG… delayed intervention group, CG…Control group**

**iPOS subscale – psychological and practical problems (professionals)**

| **iPOS subscale: psychological and practical problems (professionals)** | **tV** | | **t2** | |
| --- | --- | --- | --- | --- |
|  | **IG** | **CG** | **IG** | **CG** |
|  |  |  |  |  |
|  | N = 21 | N = 7 | N = 21 | N = 7 |
| N(missing) | 0 | 0 | 0 | 0 |
| average | 9.9 | 11.4 | 8.1 | 10.9 |
| STD | 2.6 | 3.6 | 2.7 | 3.0 |
| Min | 3 | 5 | 3 | 7 |
| Q1 | 8.0 | 8.0 | 7.0 | 7.0 |
| Median | 9.0 | 13.0 | 8.0 | 11.0 |
| Q3 | 12.0 | 14.0 | 10.0 | 14.0 |
| Max | 14 | 14 | 13 | 14 |
|  |  |  |  |  |
| Change to tV | - | - | N = 21 | N = 7 |
| N(fehlend) |  |  | 0 | 0 |
| average |  |  | -1.7 | -0.6 |
| STD |  |  | 2.6 | 1.9 |
| Min |  |  | -7 | -3 |
| Q1 |  |  | -4.0 | -3.0 |
| Median |  |  | -1.0 | 0.0 |
| Q3 |  |  | 0.0 | 1.0 |
| Max |  |  | 2 | 2 |

**Table 5: descriptive analysis of intra-individual change – iPOS subscale psychological and practical problems (professionals) – descriptive statistics, complete cases, tV… before consultation (-3 to 0 days before consultation); t2… after consultation (3 to 7 days). tV… before consultation (-3 to 0 days before consultation); t2… after consultation (3 to 7 days). IG…Intevention group, CG…Control group**

|  | | | **N** | **Estimate** | **Lower** | **Upper** | **Pr > \|t\|** |
| --- | --- | --- | --- | --- | --- | --- | --- |
| IG + del. IG | Comparison-group | IG + del. IG | 30 | -1.479 | -3.183 | 0.224 | 0.085 |
|  |  | CG | 7 | -0.055 | -3.130 | 3.021 | 0.971 |
| IG (restricted to first year) | Comparison-group | IG | 11 | -1.455 | -3.129 | 0.220 | 0.081 |
|  |  | CG | 7 | -0.571 | -2.671 | 1.528 | 0.553 |
| IG + del. IG (with consultation recommendations fully implemented) | Comparison-group | IG + verz. IG | 19 | -2.287 | -3.511 | -1.064 | 0.001 |
|  |  | CG | 7 | -0.518 | -2.501 | 1.465 | 0.582 |
| adjusted to age and baseline | Comparison-group | IG | 21 | -1.848 | -2.865 | -0.832 | 0.002 |
|  |  | CG | 7 | -0.202 | -1.954 | 1.550 | 0.808 |
| IG + del. IG; adjusted to age and baseline | Comparison-group | IG + del. IG | 30 | -1.482 | -2.797 | -0.168 | 0.029 |
|  |  | CG | 7 | -0.131 | -2.544 | 2.282 | 0.911 |
| IG (restricted to first year) adjusted to age and baseline | Comparison-group | IG | 11 | -1.574 | -2.928 | -0.220 | 0.029 |
|  |  | CG | 7 | -0.383 | -2.092 | 1.325 | 0.612 |
| IG + del. IG (with consultation recommendations fully implemented); adjusted to age and baseline | Comparison-group | IG + del. IG | 19 | -1.999 | -3.192 | -0.806 | 0.004 |
|  |  | CG | 7 | -0.615 | -2.458 | 1.229 | 0.478 |

Table 6: statistical analysis of the intervention for iPOS subscale: psychological and practical problems (professionals) – sensitivity analysis

|  | | | **N** | **Estimate** | **Lower** | **Upper** | **Pr > \|t\|** |
| --- | --- | --- | --- | --- | --- | --- | --- |
| Change - iPOS (professionals) | Comparison group | IG | 45 | -3.081 | -5.353 | -0.809 | 0.008 |
|  |  | CG | 16 | -2.273 | -6.008 | 1.463 | 0.232 |

**Table 7: Post hoc analysis, statistical analysis of intra-individual change with multiple imputations – iPOS (professionals); IG… Intervention group, CG…Control group**

|  | **Estimate** | **Lower** | **Upper** | **Pr > \|t\|** |
| --- | --- | --- | --- | --- |
| IG vs CG of Change - iPOS (professionals) | -0.809 | -5.220 | 3.603 | 0.719 |

**Table 8: Post hoc analysis, statistical analysis of the intervention for iPOS with multiple imputations – iPOS (professionals); IG… Intervention group, CG…Control group**
