# Supplementary material for: Investigating video consultations as a new form of care for neuropalliative patients in specialized outpatient care: results from the project TANNE (telemedical answers to neurological inquires in real time)
Source: Front Neurol. 2026 Apr 15;17:1730210. doi: 10.3389/fneur.2026.1730210 (PMC13126451; doi:10.3389/fneur.2026.1730210)
Supplement: Supplementary file 4 [file Data_Sheet_4.DOCX]

# Supplement 4

## Information on missing data and Baseline characteristics of Complete Case patients

**Table 1:** iPOS (Professional) – descriptive analysis of intra-individual change per event including the number of missing, tV… before consultation (-3 to 0 days before consultation); t2… after consultation (3 to 7 days).

| **iPOS (Professionals)** | **tV** | | **t2** | |
| --- | --- | --- | --- | --- |
|  | **IG** | **CG** | **IG** | **CG** |
|  |  |  |  |  |
|  | N = 25 | N = 7 | N = 25 | N = 8 |
| **N(missing)** | **20** | **9** | **20** | **8** |
| average | 28.4 | 25.6 | 25.1 | 28.4 |
| STD | 7.1 | 4.9 | 7.2 | 9.1 |
| Min | 15 | 20 | 14 | 18 |
| Q1 | 24.0 | 21.0 | 21.0 | 21.5 |
| Median | 26.0 | 26.0 | 24.0 | 26.5 |
| Q3 | 33.0 | 30.0 | 28.0 | 33.5 |
| Max | 44 | 33 | 43 | 46 |
|  |  |  |  |  |
| Change to tV | - | - | N = 16 | N = 6 |
| **N(missing)** |  |  | **29** | **10** |
| average |  |  | -2.6 | -1.3 |
| STD |  |  | 4.1 | 8.4 |
| Min |  |  | -14 | -13 |
| Q1 |  |  | -5.0 | -9.0 |
| Median |  |  | -1.5 | 0.5 |
| Q3 |  |  | -0.5 | 4.0 |
| Max |  |  | 3 | 9 |

Table **2**: Demographic data of patients accounting for complete cases; sex/team type/region/underlying disease per patient, ECOG per event (multiple events per patient possible).

|  | | | | **Comparison group and study arm** | | | |
| --- | --- | --- | --- | --- | --- | --- | --- |
|  |  | | | Intervention group (IG) | | | Control group |
|  |  | | | S1,1 | S1,2 | S1 | S2,1 |
|  | Number of patients | | | 5 | 5 | 10 | 6 |
| **Sex (n; %)** | missing | | | 0 | 0 | 0 | 1 |
|  | male | | | 0 (0) | 1 (20.0) | 1 (10.0) | 2 (40.0) |
|  | female | | | 5 (100) | 4 (80.0) | 9 (90.0) | 3 (60.0)) |
|  | | | |  |  |  |  |
| **Age average (STD)** | | | missing | 0 | 0 | 0 | 1 |
|  | | | | 56.4 (19.8) | 69.9 (9.0) | 63.0 (16.1) | 63.2 (8.0) |
|  |  | | |  |  |  |  |
| **ECOG** | missing | | | 1 | 0 | 1 | 1 |
|  | ECOG≤2 | | | 1 (25.0) | 2 (40.0) | 3 (33.3) | 2 (40.0) |
|  | ECOG>2 | | | 3 (75.0) | 3 (60.0) | 6 (66.7) | 3 (60.0) |
|  |  | | |  |  |  |  |
| **Patient per team type** | | SOPC | | 3 (60.0) | 3 (60.0) | 6 (60.0) | 6 (100) |
|  | Hospice | | | 2 (40.0) | 2 (40.0) | 4 (40.0) | 0 (0) |
|  |  | | |  |  |  |  |
| **Patient per region** | rural | | | 1 (20.0) | 2 (40.0) | 3 (30.0) | 4 (66.7) |
|  | urban | | | 4 (80.0) | 3 (60.0) | 7 (70.0) | 2 (33.3) |
|  |  | | |  |  |  |  |
| **Underlying disease** | neurological | | | 4 (80.0) | 4 (80.0) | 8 (80.0) | 6 (100) |
| neurodegenerative | | | | 2 (40.0) | 1 (20.0) | 3 (30.0) | 2 (33.3) |
