# Supplementary material for: Investigating video consultations as a new form of care for neuropalliative patients in specialized outpatient care: results from the project TANNE (telemedical answers to neurological inquires in real time)
Source: Front Neurol. 2026 Apr 15;17:1730210. doi: 10.3389/fneur.2026.1730210 (PMC13126451; doi:10.3389/fneur.2026.1730210)
Supplement: Supplementary file 5 [file Data_Sheet_5.DOCX]

# Supplement 5

|  | **Estimate** | **Lower** | **Upper** | **Pr > \|t\|** |
| --- | --- | --- | --- | --- |
| Health status of the patient (patient) | 0.902 | -1.631 | 3.436 | 0.379 |

Table 1: Statistical analysis of the intervention for health status after consultation

|  | **Estimate** | **Lower** | **Upper** | **Pr > \|t\|** |
| --- | --- | --- | --- | --- |
| IG+del. IG vs. CG | 0.863 | -1.206 | 2.933 | 0.370 |
| IG (restricted to first year) vs. CG | 1.223 | -1.440 | 3.887 | 0.271 |
| IG + del. IG (with consultation recommendations fully implemented) vs. CG | 1.350 | -0.575 | 3.275 | 0.123 |
| IG vs. KG; adjusted to age | 0.202 | -2.539 | 2.943 | 0.830 |
| IG + del. IG vs. CG; adjusted to age | 0.281 | -1.712 | 2.274 | 0.754 |
| IG (restricted to first year) vs. CG; adjusted to age | 0.432 | -2.047 | 2.911 | 0.618 |
| IG + del. IG (with consultation recommendations fully implemented) vs. CG; adjusted to age | 1.108 | -1.379 | 3.595 | 0.251 |

Table 2: Statistical analysis of the intervention for health status after consultation – sensitivity analyses.
